# An unusual case of hepatosplenic T‐cell lymphoma‐like unclassifiable T/NK‐cell lymphoma accompanied by acute myeloid leukemia

**DOI:** 10.1002/jha2.565

**Published:** 2022-09-09

**Authors:** Shin Lee, Yusuke Kito, Kei Fujita, Hiroto Wakayama, Masaki Kimura, Keisuke Kawashima, Tetsuya Tabata, Tadashi Yoshino, Takeshi Hara, Hisashi Tsurumi

**Affiliations:** ^1^ Department of Hematology and Oncology Matsunami General Hospital Gifu Japan; ^2^ Department of Pathology Matsunami General Hospital Gifu Japan; ^3^ Department of Gastrointestinal Surgery Matsunami General Hospital Gifu Japan; ^4^ Department of Pathology Okayama University Graduate School of Medicine Dentistry and Pharmaceutical Sciences, Okayama, Japan

**Keywords:** acute myeloid leukemia, CD56, hepatosplenic T‐cell lymphoma, splenomegaly, TCR rearrangement

## Abstract

We describe a case of unclassifiable T/NK‐cell lymphoma with concomitant acute myeloid leukemia (AML). A 73‐year‐old Japanese man was diagnosed with AML by bone marrow smear, but the presence of splenomegaly and liver tumor was incompatible with AML. Splenectomy and hepatic resection were performed to resolve the thrombocytopenia and define the diagnosis. The pathological findings showed sinusoidal involvement of abnormal lymphoid cells that were CD3‐positive but negative for T‐cell receptor (TCR) rearrangement. Our case could not be categorized as hepatosplenic T‐cell lymphoma because of the lack of immunohistological expression of TCR, despite the clinical similarity.

A significant advance has been made in the current World Health Organization (WHO) classification of T‐cell and natural killer (NK)‐cell neoplasms compared with the 2008 WHO classification, based largely on the approach to the genetic background [1, 2]. Hepatosplenic T‐cell lymphoma (HSTCL) is a rare subtype of non‐Hodgkin lymphomas that is characterized by involvement of the spleen, liver, and bone marrow, and presents with an aggressive clinical course [3]. Pathologically, abnormal lymphocytes infiltrate the sinusoids of organs, which are mostly T‐cell receptor (TCR)γδ‐positive [4]. Few cases of HSTCL concomitant with myeloid neoplasms have been reported [5].

## 1 CASE

**FIGURE 1:**
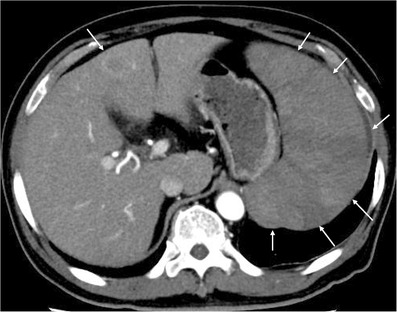
Contrast‐enhanced abdominal computed tomography (CT) reveals massive splenomegaly and a space‐occupying lesion with ring‐like enhancement in the S4 segment of the liver

A 73‐year‐old Japanese man was admitted to our hospital with malaise. His spleen was palpable 8.0 cm below the left costal margin. Laboratory data showed the following: white blood cell count, 2.0 × 10^9^/L (neutrophils, 58.0%; eosinophils, 0.0%; blasts, 1.0%); hemoglobin, 12.2 g/dl; platelet count, 44 × 10^9^/L; serum ferritin, 1,506 ng/ml; aspartate aminotransferase, 56 IU/L; alanine aminotransferase, 16 IU/L; and lactate dehydrogenase (LDH), 527 U/L. Wilms' tumor gene 1 mRNA level in peripheral blood was lower than 50 copies/μg RNA and serum soluble interleukin‐2 receptor level was 25,700 U/ml. Abdominal contrast‐enhanced computed tomography (CECT) revealed massive splenomegaly and a space‐occupying lesion (SOL) showing ring‐like enhancement in the S4 segment of the liver (Figure 1). Bone marrow smears showed a very low concentration of cells (nucleated cell count, 0.7 × 10^4^/μl) with 20.5% blasts (Figure S1A). Myeloperoxidase (MPO) staining of blasts yielded positive results on cytochemistry (Figure S1B). G‐banding analysis of bone marrow revealed a complex karyotype: 51, Y, –X, –4, –6, –7, add(11)(q2.3), –15, –22, +11mar[1/analyzed 20 cells]/46, XY[19/20]. At this point, AML was diagnosed.

**FIGURE 2:**
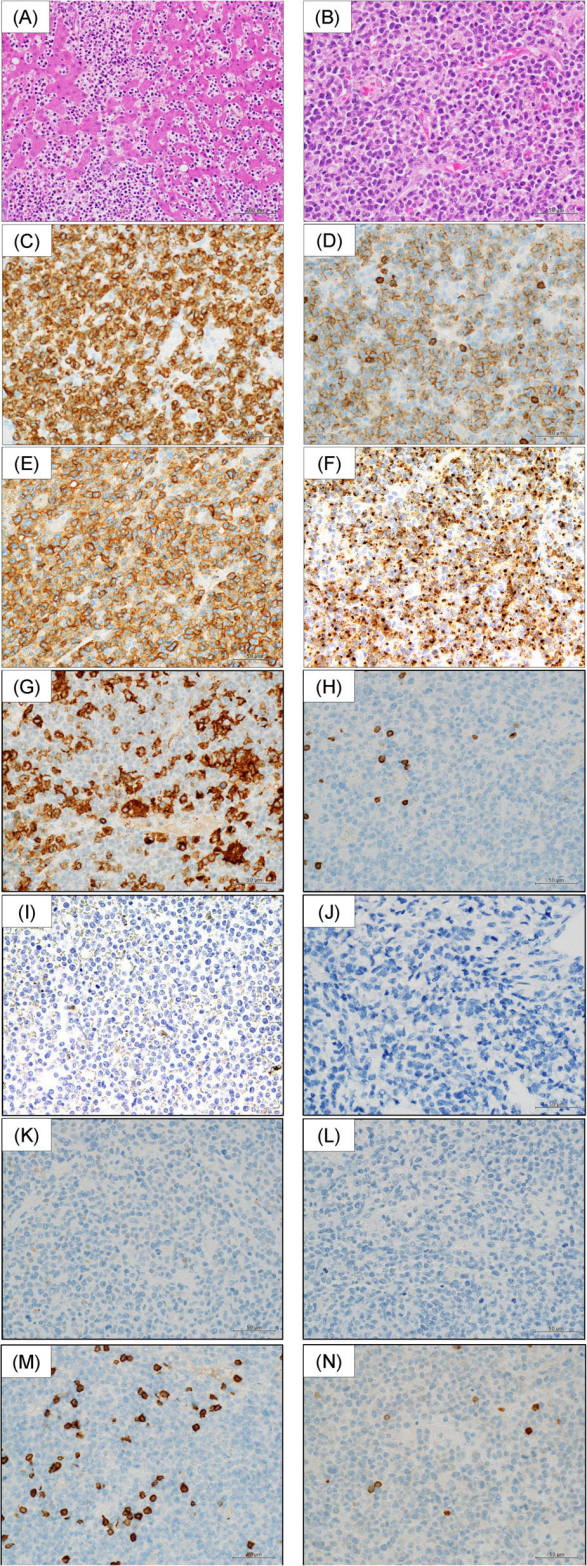
Immunohistochemical features of the liver tumor and spleen biopsy sample. Hematoxylin and eosin staining shows proliferation of medium‐sized abnormal lymphoid cells with irregular nuclei (A, × 200; B, × 400). Abnormal cells are positive for CD3 (C), CD8 (D), CD56 (E), T‐cell intracellular antigen‐1 (TIA‐1) and granzyme B (F), and negative for CD4 (G), CD5 (H), TCR‐βF1 (I), TCR‐CγM1 (J), MPO (K), EBER‐ISH (L), CD20 (M), and CD79a (N)

Bone marrow biopsy was performed at the start of induction therapy for AML (AV therapy; continuous drip infusion of low dose cytarabine, 20 mg/body and etoposide, 50 mg/body for 10 days) on suspicion of a lymphoid tumor complication [6]. Bone marrow polymerase chain reaction (PCR) analysis of immunoglobulin heavy chain (IgH) and TCR gene rearrangements yielded negative results for IgH, but positive results for Vβ‐Jβ2 and Dβ‐Jβ2 rearrangements. Bone marrow biopsy revealed high cellularity (90%) and moderate fibrosis. Proliferation was seen for CD3‐positive, medium‐sized, abnormal lymphoid cells with irregular nuclei that were also positive for CD8 and CD34, and negative for CD5, CD79a, CD30, and EBV‐encoded small RNA‐1 in situ hybridization (EBER‐ISH) (Figure 2). The pathological diagnosis from bone marrow biopsy was bone marrow involvement of T‐cell lymphoma. The simultaneous presence of two different hematological malignancies was demonstrated, and T‐cell lymphoma accompanied by AML was diagnosed.

Oral prednisolone (PSL) was started at 40 mg/day on day 6 of induction therapy for transfusion‐refractory thrombocytopenia due to massive splenomegaly. Spleen size was markedly reduced on palpation, and platelet transfusion reactivity was recovered. However, transfusion‐refractory thrombocytopenia recurred on day 19 of induction therapy, and the recurrence of splenomegaly and multiple SOLs in the liver was detected on abdominal CECT on day 24 of induction therapy. On day 25 of induction therapy, laparoscopic‐assisted splenectomy and hepatic resection were performed to resolve the thrombocytopenia and reach a definitive diagnosis. Although thrombocytopenia resolved after the surgery, serum LDH level rapidly elevated. To control lymphoma, we administrated methylprednisolone at 500 mg/day, followed by CHOP therapy (50 mg/m^2^ of doxorubicin, 750 mg/m^2^ of cyclophosphamide, 1.4 mg/m^2^ of vincristine on day 1, and 100 mg/body of PSL on days 1–5) from postoperative day 4. However, the clinical condition of the patient became progressively worse and he died on day 10 of CHOP due to lymphoma.

After pathological consultation, the final diagnosis was unclassifiable T/NK‐cell lymphoma accompanied by AML. Findings from the liver and spleen were the same, comprising sinusoidal involvement of the CD3‐positive abnormal cells with high Ki‐67 expression, which were positive for CD8, CD56, T‐cell intracellular antigen‐1(TIA‐1), and granzyme B and negative for CD4, CD5, TCR‐βF1, TCR‐CγM1, MPO, EBER‐ISH, and CD79a (Figure 2). MPO‐positive myeloblasts were scattered in the lymphoma background.

## 2 DISCUSSION

We have described the case of a patient diagnosed with unclassifiable T/NK‐cell lymphoma accompanying AML with features of splenomegaly, liver, and bone marrow involvement without lymphadenopathy. Our case resembled HSTCL based on the clinical and pathological features, but the lack of TCR rearrangement on immunostaining does not fit the HSTCL category. Both induction therapy for AML and CHOP therapy proved ineffective, resulting in early death of the patient.

The pattern of involvement, pathological, and genetic features with TCR‐β rearrangement detected by PCR suggest this case is close to HSTCL [3]. The detection of TCR‐β rearrangement by PCR represents a point of difference from the majority of HSTCL with TCR‐γδ rearrangement [7]. In our case, expression of CD3, CD2, and TIA‐1 and the absence of CD4, CD5, and EBER‐ISH were all compatible with HSTCL [4, 8]. CD8 and CD56 can occasionally be expressed in HSTCL [4, 8]. The biggest discrepancy in the diagnosis of HSTCL in our case was the lack of expression of TCR rearrangement on immunohistochemistry [8]. TCR‐γ and TCR‐β gene rearrangement studies are insufficient to define the origin of lymphomatous neoplasms because of the occasional detection of non‐productive γ‐ or β‐chain rearrangements [9]. As a result, our case cannot be diagnosed as αβ HSTCL. No previous reports have described concomitant HSTCL and AML, but a case of concomitant αβ HSTCL and myelodysplastic syndrome has been reported [5]. In the background of the lymphoma, infiltration of myeloid blasts was observed in bone marrow, the liver, and the spleen. We considered that the involvement of leukemic blasts in organs was based on the simultaneous presence of two different neoplasms. Because of the diagnostic difficulties and fatal outcome, a series of similar cases should be accumulated to help in the development of future diagnostics.

## AUTHOR CONTRIBUTIONS

Shin Lee, Yusuke Kito , and Hisashi Tsurumi contributed to the conception and the design of the study. Shin Lee and Yusuke Kito contributed to the literature review and the writing of the manuscript. Kei Fujita, Hiroto Wakayama, Keisuke Kawashima, and Hisashi Tsurumi contributed to the revision of the manuscript. Kei Fujita contributed to figure creation. Tadashi Yoshino contributed to the pathological diagnostic process and the manuscript writing. Shin Lee confirms the authenticity of all the raw data. All authors approved the final manuscript version submitted for publication.

## CONFLICT OF INTEREST

The authors declare they have no conflicts of interest.

## FUNDING STATEMENT

The authors received no specific funding for this work.

## ETHICS STATEMENT

In accordance with our institution's ethics board, written consent forms were not required, as less than 10 cases are reported.

## Supporting information

Supporting Information

## Data Availability

No patient identifiable information was submitted.
