# Supplementary figures and images for: An unusual case of hepatosplenic T‐cell lymphoma‐like unclassifiable T/NK‐cell lymphoma accompanied by acute myeloid leukemia
Source: EJHaem. 2022 Sep 9;3(4):1335–8. doi: 10.1002/jha2.565 (PMC9713219; doi:10.1002/jha2.565)

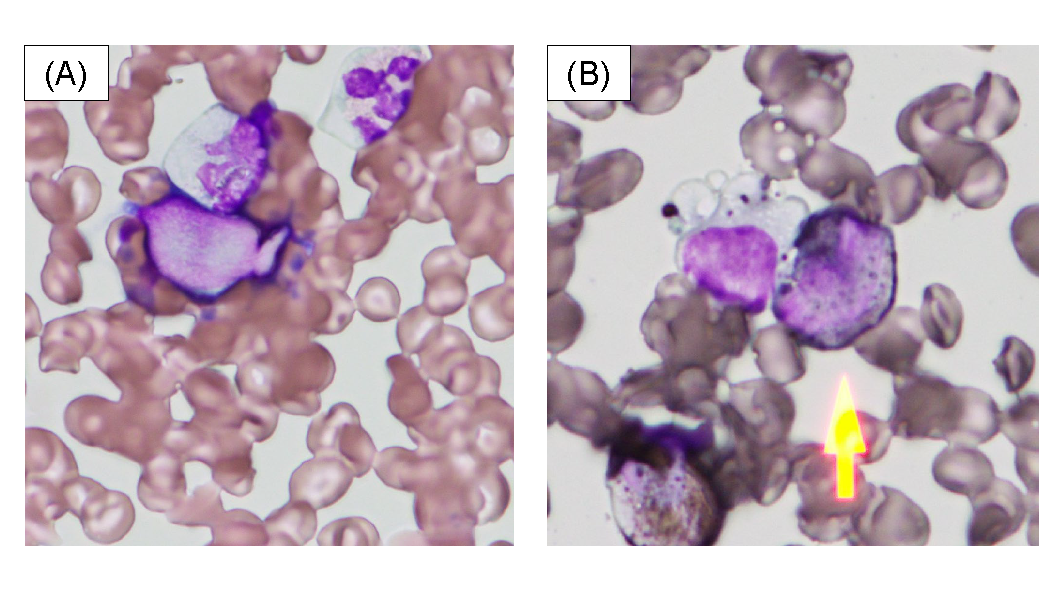

Supplement: Supplementary file 1 — Supporting Information [file JHA2-3-1335-s001.tif]
